# Association Between Leptin (-2548G/A) Genes Polymorphism and Breast Cancer Susceptibility: A Meta-Analysis

**DOI:** 10.1097/MD.0000000000002566

**Published:** 2016-01-29

**Authors:** Wanjun Yan, Xingcong Ma, Xiaoyan Gao, Shuqun Zhang

**Affiliations:** From the Department of Oncology, the Second Affiliated Hospital, Xi’an Jiaotong University, Xi’an, Shaanxi, P.R. China.

## Abstract

Leptin is a confirmed breast cancer susceptibility gene. However, published studies reported mixed results. This meta-analysis was conducted to systematically get a more accurate estimation of the association between the Leptin (-2548G/A) gene polymorphism and breast cancer risk.

To assess the effect of Leptin (-2548G/A) gene polymorphism on breast cancer susceptibility, we searched PUBMED, ISI Web of Knowledge, EMBASE, Chinese National Knowledge Infrastructure (CNKI) databases until September 2015 to identify eligible studies, without restriction. Summary odds ratios (ORs) and 95% confidence intervals (CIs) were used to evaluate the susceptibility to breast cancer. Separate analyses were conducted on features of the population such as ethnicity, source of controls, and country.

A total of 9 case-control studies on Leptin (-2548G/A) gene polymorphism and breast cancer risk, including 3725 cases and 3093 case-free controls were identified. The results revealed that compared with the G allele, the A allele was associated with modestly increased risk of overall breast cancer (A vs G: OR = 1.12, 95%CI = 1.04 − 1.20, *P* = 0.002, *P*_het_*P* < 0.00001). Following further stratified analyses, in the subgroup analyses by ethnicity, a significantly increased risk was observed among Caucasian (A vs G: OR = 1.11, 95%CI = 1.03–1.20, *P* = 0.006, *P*_het_ = 0.00001). No publication bias was found in the present study.

In conclusion, our meta-analysis suggests that the Leptin (-2548G/A) gene polymorphism plays an important role in breast cancer susceptibility, especially in Caucasian.

## INTRODUCTION

Breast cancer is the most frequently diagnosed cancer among women,^1^ but its etiology is still not fully understood. It is a heterogeneous disease in regard to its clinical, histological, and molecular profile.^2–4^ At present, genetic polymorphisms are increasingly recognized as contributors to breast cancer risk.^5^

Leptin, a 16 kD polypeptide hormone encoded by the obese gene,^6^ plays an important role in body weight homeostasis through effects on food intake and energy expenditure.^7–8^ Obesity has been identified as a risk factor for breast cancer in postmenopausal women,^9–11^ and increased leptin levels have been frequently found in obese subjects, with higher expression in women relative to men.^12–14^ Moreover, studies have indicated that the leptin is involved in carcinogenesis of breast tissue and acts to favor the proliferation, angiogenesis, progression, and poor survival of breast cancer cells,^15–17^ especially in higher grade tumors and are associated with distant metastasis. Delort and coworkers^18^ suggested that the multifaceted role of leptin in breast cancer development and the different molecular pathways involved such as inflammation, oxidative stress, and antitumor immunity. Further, over expression of leptin in breast cancer appears to be associated with higher tumor grade and size.^19–23^ These data suggest that leptin is likely to play a key role in carcinogenesis. However, the mechanisms of leptin and tumorigenesis still remain poorly understood.

In recent years, most studies concern leptin gene polymorphism involvement in breast cancer, including rs2060713C/T, rs7799039, G-2548A, G-223A, G-2549A. Interestingly, G-2548A is a rather popular polymorphism and focus on studying breast cancer. The A allele of -2548G/A polymorphism was found to be associated with higher leptin levels before lower BMI (body mass index) loss in women.^24^ The functional influence of the -2548G/A polymorphism on leptin expression stimulates great interest of many investigators to examine its association with breast cancers.^25–31^ However, only a few studies with inconsistent results have addressed the relationship between leptin and breast cancer.^32–39^ Liu and coworkers^40^ suggested that the A allele of -2548G/A polymorphism may be a determinant of cancer development. However, it was not concentrated on studying breast cancer. A meta-analysis of Niu et al^41^ suggested that the leptin level plays a role in breast cancer and has potential for development as a diagnostic tool. Therefore, we performed a meta-analysis of early-release publications to clarify whether the -2548G/A polymorphism acts on breast cancer susceptibility in the first place.

## MATERIALS AND METHODS

### Publication Search

Computer searches were carried out independently by 2 authors, in PubMed, Web of Knowledge, Embase, and the Chinese National Knowledge Infrastructure (CNKI) database (last search: August 10, 2015) to collect articles with case-control studies related to the association of the Leptin (-2548G/A) gene polymorphism and breast cancer risk.

The keywords were as follows: breast cancer/carcinoma/neoplasm/tumor, Leptin (-2548G/A), variant/genotype/polymorphism/SNP. Furthermore, reference lists of main reports and review articles were also reviewed by a manual search to identify additional relevant publications. Searches were limited to papers published in English only. The study was approved by the Conduct of Human Ethics Committee of the Second Affiliated Hospital, Xi’an Jiaotong University.

### Selection and Exclusion Criteria

Two investigators independently screened all titles and abstracts of the identified studies. Studies included should be in accordance with the following criteria: (1) a case-control study with matching control subjects; (2) clinical trials studying the association between Leptin (-2548G/A) gene polymorphisms and breast cancer risk; (3) all cases were diagnosed by the pathological examination; (4) contained at least 2 comparison groups (cancer group and control group); (5) reported available genotype frequencies dates for both patient and control populations. Accordingly, the following exclusion criteria were also used: (1) no controls; (2) the source of cases and controls, and other essential information were not provided; (3) no sufficient data reported; (4) abstracts, comments, reviews, meta-analysis, and duplicated publications.

### Data Extraction and Synthesis

All the available data were extracted from each study by 2 of the authors independently, and then discussed by the research team. For each included study, the following information was collected: the first author, year of publication, country of origin, ethnicity, source of control, numbers of cases and controls, and numbers of GG, GA and AA genotypes in cases and controls. Different ethnic ancestries were categorized as Caucasian, and “mixed”. The “mixed” group means mixed or unknown populations. All the case and control groups were well controlled. The non-cancer controls had no history of gynecologic disease, and there was no present evidence of gynecologic cancer, any malignant disease, or genetic disease, and there was no present evidence of any malignant disease. When studies included subjects of >1 ethnicity, genotype data were extracted separately according to ethnicities for subgroup analyses.

### Statistical Analysis

We carried out this meta-analysis referring to PRISMA Guideline. The associations between the Leptin (-2548G/A) gene polymorphism and breast cancer risk were measured by odds ratios (OR) with 95% confidence intervals (CI) for A vs G, AA vs GG, AA + GA vs GG, AA vs GA + GG, and GA vs GG genetic models. Cochran *Q* test and *I*^2^ statistic were used to measure the extent of inconsistency among the results. Heterogeneity was considered significant if *P*_het_ ≤ 0.1 or *I*^2^ > 50 %. When significant heterogeneity existed, we selected a random effects model for statistics. Otherwise, a fixed effects model was used. Sensitivity analysis was also tested by removing 1 study at a time to calculate the overall homogeneity and effect size. Publication bias were evaluated by funnel plots and further assessed by Egger's linear regression test.

All statistical analyses were carried out with the review manager (RevMan 5.2 The Cochrane Collaboration, Oxford, UK) and the Stata software version 10 (Stata Corporation, College Station, TX). All *P* values in the meta-analysis were 2-sided, and *P* values <0.05 were considered significant.

## RESULTS

### Characteristics of Studies

**FIGURE 1:**
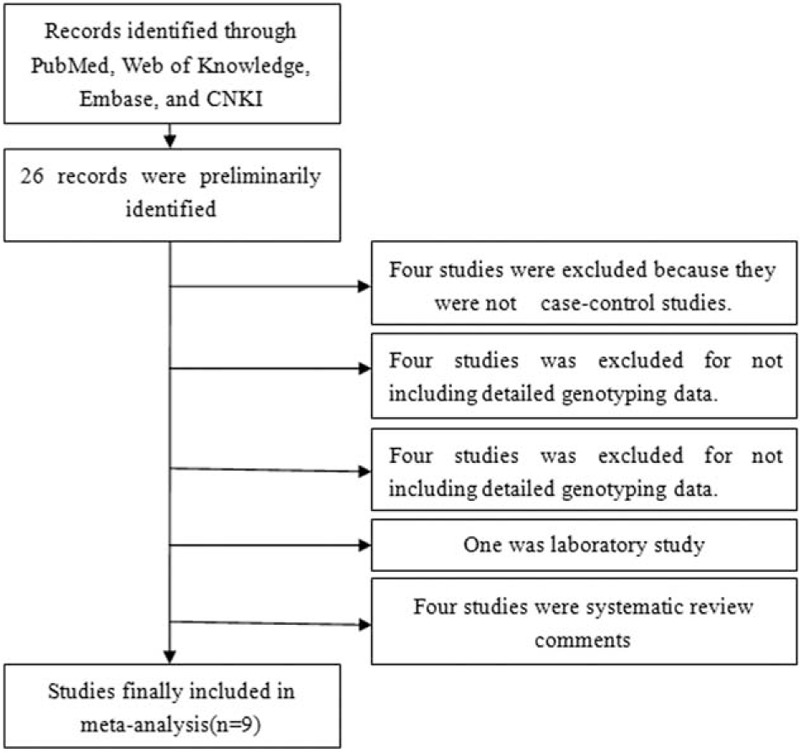
Flowchart of study selection. CNKI = Chinese National Knowledge Infrastructure.

By reading the relevant articles, there are 26 studies to identify preliminarily for further detailed evaluation. The results of excluding 17 irrelevant studies, for example: 4 studies were not case-control studies, 4 studies were not focused on Leptin (-2548G/A) gene polymorphism, 4 studies did not have genotyping data, 4 studies were review articles (Figure 1). Ultimately, a total of 9 case-control studies, including 3725 cases and 3093 case-free controls, were identified.^27,32–39^ The characteristics of 9 studies are presented in Tables 1 and 2. Breast cancers were confirmed by histology or pathology in most studies. Among them, 7 studies were based on Caucasian (America, Iran, and Greece). Two were based on Mixed (México and Tunisia).

**TABLE 1:**
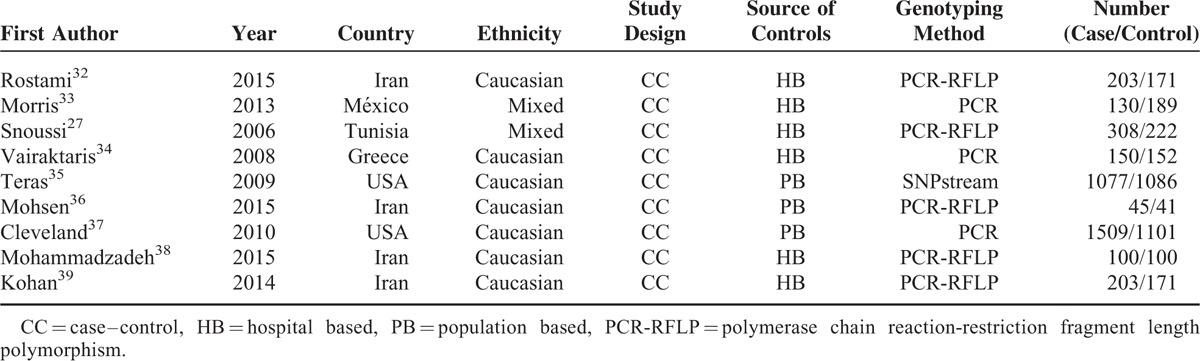
Characteristics of the Studies Included in the Meta-Analysis

**TABLE 2:**
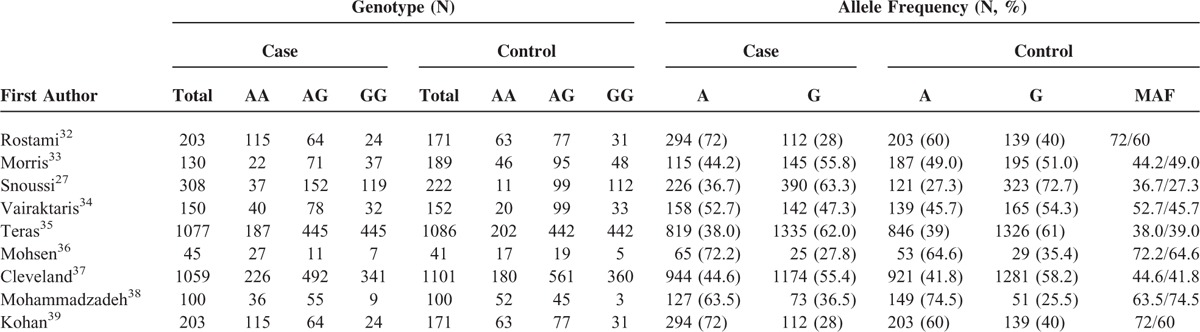
Leptin (-2548G/A) Gene Polymorphism Genotype Distribution and Allele Frequency in Cases and Controls

### Meta-Analysis Results

The frequency of the A allele varied widely across the 12 studies, ranging from 0.36 to 0.72 (Table 2). The average frequency of the A allele in overall populations was 0.55, which was lower than that in European populations (0.59). There was no significant difference between Caucasians and Mixed (*P* > 0.05).

**FIGURE 2:**
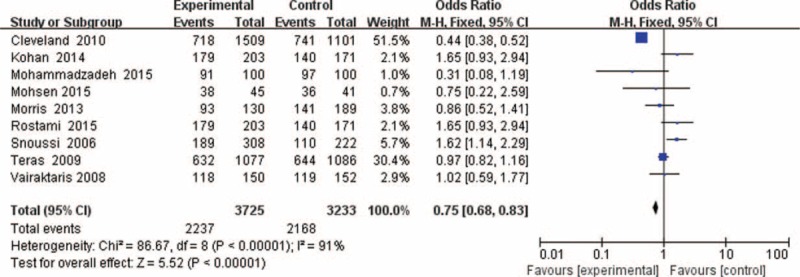
Forest plots of Leptin (-2548G/A) gene polymorphism and breast cancer risk in the overall population (AA + GA vs GG). The squares and horizontal lines correspond to the study-specific OR and 95% CI. The area of the squares reflects the weight (inverse of the variance). The diamond represents the summary OR and 95% CI.CI = confidence interval, OR = odds ratio.

The main results of this meta-analysis are listed in Table 3 and clinicopathological parameters and habits in Table 4. Overall, there was evidence of an association between cancer risk and the variant genotypes in different genetic models when all the eligible studies were pooled into the meta-analysis. As show in Table 3 and Figure 2, significant main effects were observed in 3 genetic models (A vs G: OR = 1.12, 95%CI = 1.04 − 1.20, *P* = 0.002, *P*_het_*P* < 0.00001; AA vs GG: OR = 1.23, 95%CI = 1.06 − 1.42, *P* = 0.005, *P*_het_ = 0.0001; AA + GA vs GG: OR = 0.75, 95%CI = 0.68 − 0.83, *P* < 0.00001, *P*_het_ = 0.00001). However, there were no significant associations between the Leptin (-2548G/A) gene polymorphism and breast cancer risk in other genotype distributions (GA vs GG: OR = 0.99, 95%CI = 0.88 − 1.11, *P* = 0.88, *P*_het_ = 0.39; AA vs GA + GG: OR = 1.12, 95%CI = 1.00 − 1.27, *P* = 0.06, *P*_het_ = 0.00001). Furthermore, when stratified by clinico-pathological parameters of breast cancer, there were no associations between Leptin (-2548G/A) genes and the parameters in the dominant model, such as menopausal status (OR = 0.17; 95% CI = 0.14–0.20; *P* = 0.06), clinical stage (OR = 0.24; 95% CI = 0.17–0.33; *P* = 0.21), ER/PR receptor (OR = 0.30; 95% CI = 0.22–0.41; *P* = 0.15), and lymph nodes (OR = 0.39; 95% CI = 0.28–0.53; *P* = 0.32). However, there were significant associations between Leptin (-2548G/A) genes and the parameters in the dominant model, such as BMI (OR = 0.13; 95% CI = 0.09–0.19; *P* = 0.01).

**TABLE 3:**
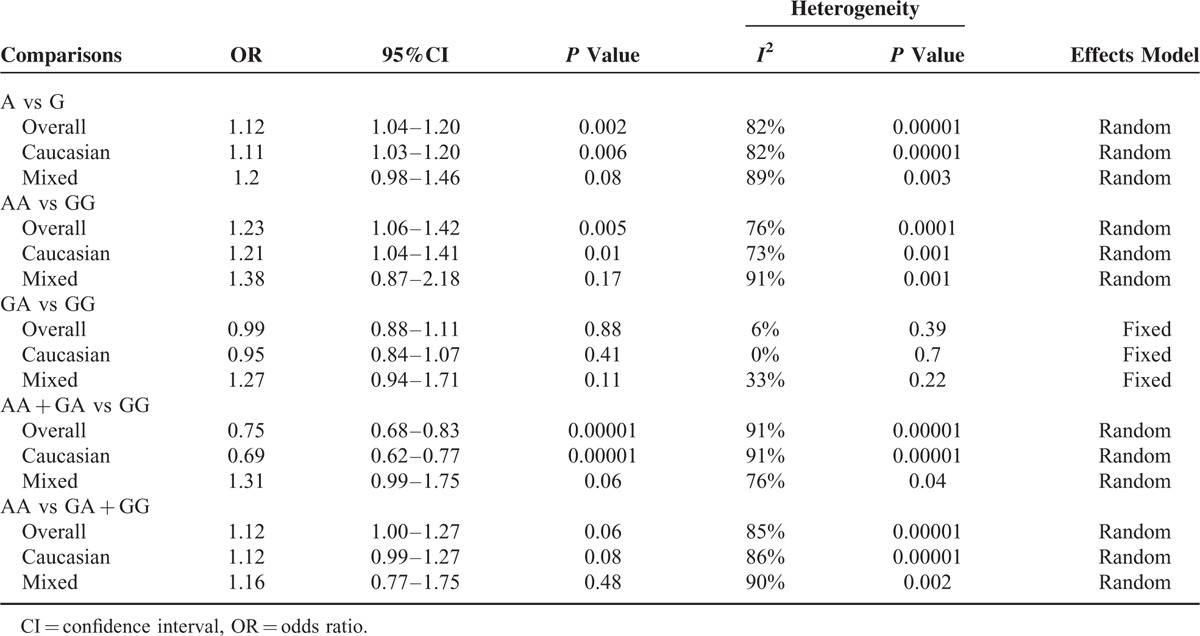
Meta-Analysis of the Association Between the Leptin (-2548G/A) Gene Polymorphism and Breast Cancer Risk

**TABLE 4:**
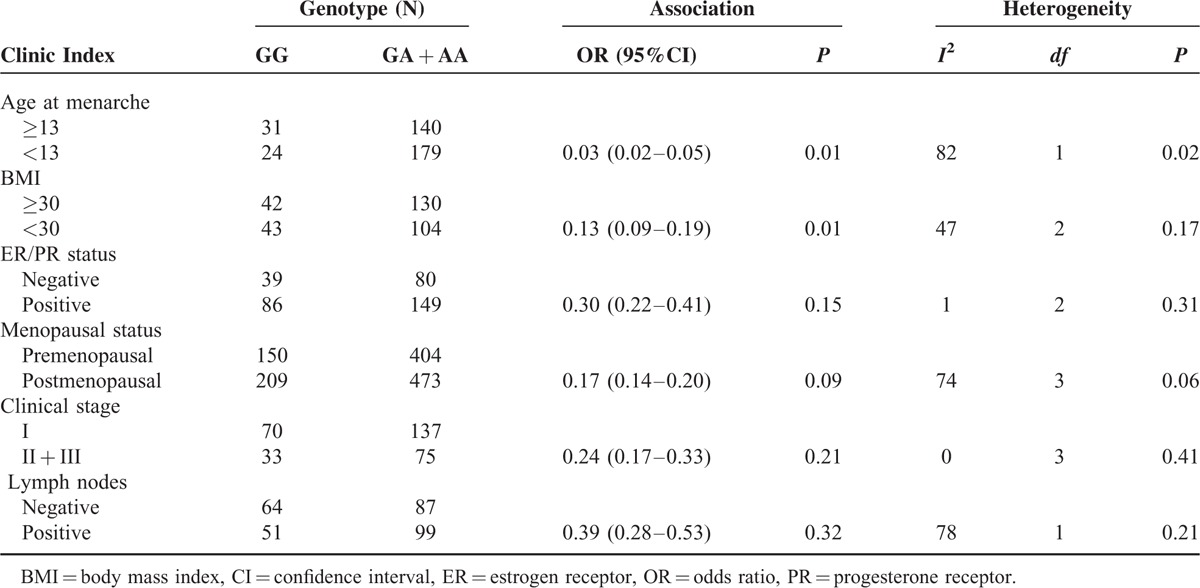
Association Between Leptin (-2548G/A) Gene Polymorphism and Clinico-Pathological Parameters and Habits of the Breast Cancer Patients Based on the Dominant Models

**FIGURE 3:**
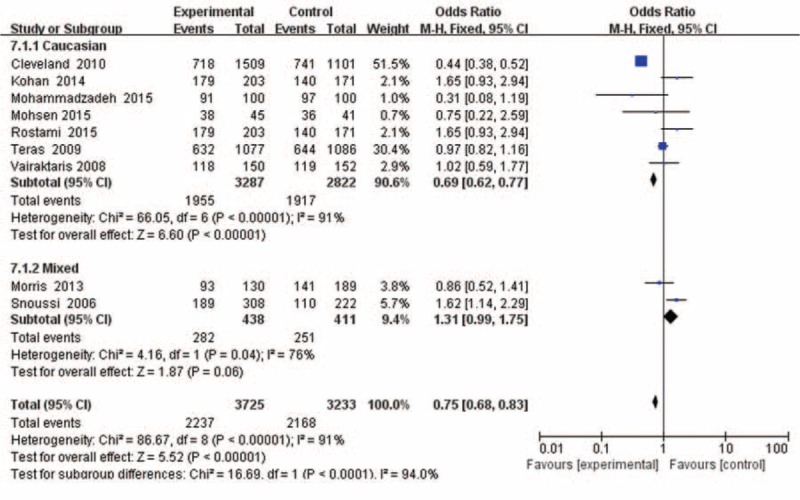
Forest plots of Leptin (-2548G/A) gene polymorphism and breast cancer risk in the Asian and Caucasian population (AA + GA vs GG). The squares and horizontal lines correspond to the study specific OR and 95% CI. The area of the squares reflects the weight (inverse of the variance). The diamond represents the summary OR and 95% CI.CI = confidence interval, OR = odds ratio.

Seven articles, including 3278 cases and 2682 controls, were used to investigate the association of the Leptin (-2548G/A) gene polymorphism with breast cancer susceptibility in Caucasians. The results showed (Figure 3) that the Leptin (-2548G/A) gene polymorphism was associated with breast cancer risk in 3 genotypes (A vs G: OR = 1.11, 95%CI = 1.03–1.20, *P* = 0.006, *P*_het_ = 0.00001; AA vs GG: OR = 1.21, 95%CI = 1.04–1.41, *P* = 0.01, *P*_het_ = 0.001; AA + GA vs GG: OR = 0.69, 95%CI = 0.62–0.77, *P* < 0.00001, *P*_het_ = 0.00001), but no associations in other genetic models (GA vs GG: OR = 0.95, 95% CI = 0.84–1.07, *P* = 0.41, *P*_het_ = 0.70; AA vs GA + GG: OR = 1.12, 95% CI = 0.99–1.27, *P* = 0.08, *P*_het_ = 0.00001).

### Publication Bias

**FIGURE 4:**
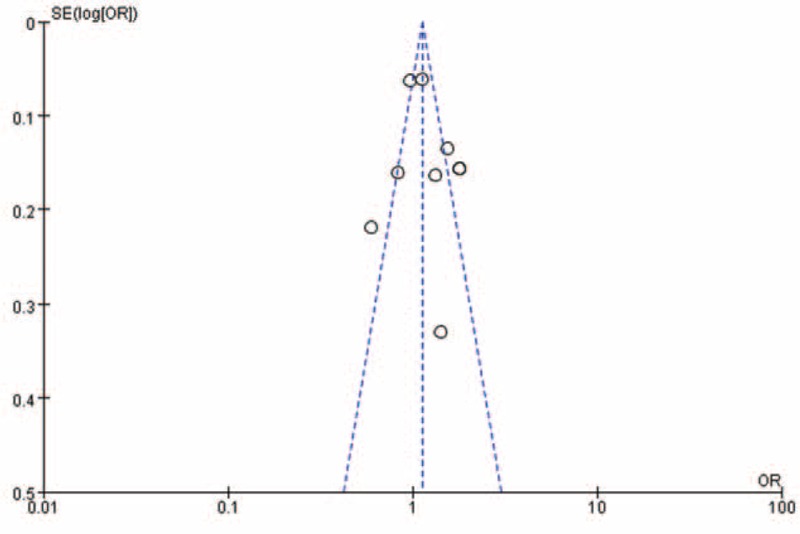
Funnel plot assessing evidence of publication bias from 9 studies (AA + GA vs GG).

Begg's funnel plot and Egger's test were performed to assess the publication bias. As show in Figure 4, the funnel plots did not reveal any obvious asymmetry in all genotypes in overall population, and the results of Begg's test revealed no publication bias (*P* > 0.05).

## DISCUSSION

Breast cancer is one of the most common malignant tumors and leading causes of cancer-related death among women in the world, and it is a threat to women's health. Terrasi and his colleagues studies have manifested that Leptin is overexpressed in human breast tumors.^42^ The Leptin (-2548) A allele, which result in high leptin secretion, was associated with increased risk of breast cancer. A leptin G-2548A gene polymorphism in the promoter region of leptin gene has been shown to correlate with variations in serum leptin levels, degree of obesity, as well as cancer susceptibility and has been shown in several reports to promote tumor growth.^43,44^ A broad range of investigations have demonstrated strong evidence for an involvement of leptin in breast cells proliferation stimulation, apoptosis inhibition, cell migration, and angiogenesis.^45–48^

A recently published meta-analysis demonstrated that the Leptin-2548G/A gene polymorphism was associated with overall cancer. In our meta-analysis, we combined all publications addressing the association of the Leptin -2548G/A gene polymorphism and breast cancer risk before August 10, 2015, and the results revealed a significantly elevated breast cancer risk, we pooled all 9 eligible case–control studies to estimate the breast cancer risk of the Leptin-2548G/A genes. We found that Leptin-2548G/A A > G variant had a significantly increased risk of breast cancer in overall population.

In the subgroup meta-analysis based on ethnicity, compared with G allele, a significantly increased breast cancer risk is associated with A allele in Caucasian population. We stratified between Leptin (-2548G/A) gene polymorphism and clinico-pathological parameters and habits of the breast cancer patients based on the dominant models. Furthermore, compared with AA genotype, a significantly increased risk of breast cancer is associated with GG genotype, the combined AG/GG genotypes subgroup. However, there were 9 studies based on Caucasian background and Mixed among the eligible studies, and there was no study based on Asians background among the eligible studies. Hence, need to further investigations on a large scale on Asians and still need to further investigations on a large scale on Caucasian populations are needed to verify this result.

Some limitations in this meta-analysis must be addressed. First, the detailed individual information in some studies was unknown; thus, we could not assess the risk of breast cancer according to stratification of environment factors, and other risk factors of cancer. Second, we considered the comparison HER2 (human epidermal growth factor receptor-2) and Leptin case subjects, in a similar fashion HER2 case subjects. But 7 of 9 references did not list some stratified data of HER2. Hence, we cannot continue to further analysis and sorting data. Moreover, further large-scale multicenter studies with more detailed individual data, with different environmental background are warranted to further validated gene–gene and gene–environment interactions on Leptin-2548G/A gene polymorphism and breast cancer risk.

## CONCLUSIONS

In summary, our present meta-analysis provides evidence of the association between Leptin-2548G/A gene polymorphism and breast cancer risk, and suggests that Leptin-2548G/A gene polymorphism has an increased risk with breast cancer. Further studies based on different ethnicity and various cancer types are warranted to verify our findings.
