# Association Between Leptin (-2548G/A) Genes Polymorphism and Breast Cancer Susceptibility: A Meta-Analysis: Erratum

**DOI:** 10.1097/01.md.0000494750.60057.7d

**Published:** 2016-08-26

In the article “Association Between Leptin (-2548G/A) Genes Polymorphism and Breast Cancer Susceptibility: A Meta-Analysis”,^1^ which appeared in Volume 95, Issue 4 of *Medicine*, the corresponding author's address was originally listed incorrectly. The article has since been corrected online.
